# Identification of a Type-Specific Epitope in the ORF2 Protein of Duck Astrovirus Type 1

**DOI:** 10.3390/ani9121069

**Published:** 2019-12-02

**Authors:** Jingjing Lan, Ruihua Zhang, Pengfei Li, Junhao Chen, Zhijing Xie, Shijin Jiang

**Affiliations:** 1 College of Veterinary Medicine, Shandong Agricultural University, Taian 271000, China; jjlan1024@163.com (J.L.); ruirui041127@126.com (R.Z.); p.li@erasmusmc.nl (P.L.); junhao-chenyy@163.com (J.C.); xiezhj@sdau.edu.cn (Z.X.); 2 Shandong Provincial Key Laboratory of Animal Biotechnology and Disease Control and Prevention, Taian 271000, China; 3 College of Public Health and Management, Weifang Medical University, Weifang 261042, China

**Keywords:** DAstV-1, mAb, ORF2 protein, B-cell epitope

## Abstract

**Simple Summary:**

Duck astrovirus type 1 (DAstV-1) infection constitutes a cause of viral hepatitis in ducklings and little is known about the B-cell epitope of DAstV-1. In this study, using a monoclonal antibody (mAb) 3D2 against ORF2 protein of DAstV-1, a highly conserved linear B-cell epitope of ^454^ STTESA^459^ in DAstV-1 ORF2 was identified. The mAb 3D2 showed no neutralizing activity to DAstV-1 and had no cross-reactivity with other DAstV serotypes.

**Abstract:**

Duck astrovirus type 1 (DAstV-1) infection constitutes a cause of viral hepatitis in ducklings and little is known about the B-cell epitope of DAstV-1. In this study, a monoclonal antibody (mAb) 3D2 against open reading frame 2 (ORF2) protein of DAstV-1 was used to identify the possible epitope in the four serotypes of DAstV. The mAb 3D2 showed no neutralization activity to DAstV-1, and reacted with the conserved linear B-cell epitopes of ^454^STTESA^459^ in DAstV-1 ORF2 protein. Sequence analysis, dot blot assay, and cross-reactivity test indicated that the epitope peptide was highly conserved in DAstV-1 sequence and mAb 3D2 had no cross-reactivity with other DAstV serotypes. To the best of our knowledge, this is the first report about identification of the specific conserved linear B-cell epitope of DAstV-1, which will facilitate the serologic diagnosis of DAstV-1 infection.

## 1. Introduction

Astroviruses (AstVs) are nonenveloped, single-stranded, positive-sense RNA viruses whose name comes from ‘astron’ [1]. The *Astrovitidae* family includes two genera of *Mamastrovirus* (MAstV) and *Avastrovirus* (AAstV), causing infection in mammalian and avian species, respectively [2]. Although MAstVs have been considered as enteric pathogens usually with mild and self-limiting characteristics in mammals [3,4,5,6], it had also been reported that MAstV could cause serious disease such as encephalitis in different species [7,8,9]. In terms of AAstV, it could induce severe disease to poultry, such as poultry mortality syndrome and enteritis in turkeys [10,11], acute nephritis in chickens [12], fatal hepatitis in young ducklings [13], and fatal visceral gout in goslings [14]. 

Duck astrovirus (DAstV) was divided into four serotypes: DAstV-1 [13], DAstV-2 [15], and the newly found DAstV-3 [16] and DAstV-4 [17]. DAstV-1 disease has spread worldwide and continued to threaten the duck industry because of the symptom of fatal hepatitis in young ducklings [13,18]. The genome of DAstV-1 is 6.4–7.9 kb in length, comprising of three open reading frames (ORF1a, 1b, and 2), 5′ and 3′ untranslated region (UTR), and a poly A tail [19]. Both the ORF1a and ORF1b encode the nonstructural proteins (NSPs), containing enzymes and participating in viral replication, whereas the ORF2 encodes the viral capsid polyprotein [20,21]. ORF2, containing antigenic determinant, can induce the production of neutralizing antibody interacting with the host [22,23,24]. 

It is recognized that monoclonal antibodies (mAbs) consisting of one specific antibody molecule are superior to their polyclonal antisera in many facets of immunology [25,26,27]. Their characteristics of sensitive and specificity make hybridoma-derived antibodies the effective immunological reagents in immunoassays, immunotherapy, immunoaffinity chromatography and immune diagnosis. Until now, the application of mAb in DAstV diagnosis has not been reported. In this study, taking the prokaryotic-expressed ORF2 protein as the immunogen, a DAstV-1 ORF2-specific mAb 3D2 was generated using cell hybridization technique, and a highly conserved B-cell epitope in DAstV-1 ORF2 protein was identified with the mAb. These findings will be valuable for developing epitope-based diagnostic kit for DAstV-1 infections.

## 2. Materials and Methods 

### 2.1. Viruses, Cells, and Antibodies 

DAstV-1 virulent strain D51 (GenBank accession no. MH712856) was isolated from the liver of sick cherry valley ducks in the Shandong province of China in 2012 [28]. The *ORF2* gene of DAstV-1 D51 strain was cloned into the prokaryotic expression vectors pET-32a (+) (Novagen, Darmstadt, Germany) and pGEX-6p-1 (GE Healthcare, Amersham, UK) to generate recombinant histidines tagged ORF2 (His-ORF2) and glutathione S-transferase tagged ORF2 (GST-ORF2). The purified His-ORF2 protein was used to immunize BALB/c mice. The hybridoma cell line producing mAb 3D2 was produced by fusion of B-lymphocytes from immunized mice with mouse myeloma cells. Subtype identification revealed that mAb 3D2 was of the IgG2b/kappa type. 

Horseradish peroxidase (HRP) labeled goat anti-mouse antibody and fluorescein isothiocyanate (FITC) labeled goat anti-mouse antibody were purchased from KPL (Gaithersburg, MD, USA). The positive anti-DAstV-1 serum was obtained from five mice immunized with purified His-ORF2 protein and stored in the veterinary molecular etiology laboratory of Shandong Agricultural University.

The baby hamster kidney (BHK-21) cells and duck embryo fibroblasts (DEF) cells were cultured in Dulbecco’s modified Eagle’s medium (DMEM) containing 10% fetal bovine serum (FBS) at 37 °C in a 5% CO_2_ atmosphere. A DNA-launched infectious clone of DAstV-1 D51 strain, named pABX-D51, was constructed and stored in our lab [28]. The complete *ORF2* genes of DAstV-2 SL1 strain (AHX26592), DAstV-3 CPH strain (AID55207), and DAstV-4 YP2 strain (AIS22433), and all the primers used in this study were synthesized by Sangon Biotech Co., Ltd. (Shanghai, China). 

### 2.2. Reactivity Analysis of the mAb 3D2

The complete *ORF2* genes of DAstV-1 D51 strain, DAstV-2 SL1 strain, DAstV-3 CPH strain and DAstV-4 YP2 strain were, respectively, cloned into plasmid pGEX-6p-1 with the primers in Table 1. These positive recombinant plasmids were then transformed into *Escherichia coli* BL21 (DE3) cells and induced expression using isopropyl β-d-thiogalactoside (IPTG). All the GST-fusion ORF2 proteins were purified using glutathione resins (Genscript, Piscataway, NJ, USA) and subsequently analyzed by dot blot assay according to the previous study [29]. Briefly, approximately 2.0 μg GST-fusion proteins were spotted onto the center of the nitrocellulose membrane grid, respectively. After being blocked with 5% BSA in TBST buffer (20 mM Tris-HCl, 150 mM NaCl, 0.01% Tween-20, pH7.5) for 1 h, membranes were respectively incubated with mAb 3D2 (dilution with 1:1000 in PBS), anti-GST antibody (dilution with 1:4000 in PBS), and anti-DAstV-1 sera (dilution with 1:500) at 37 °C for 1 h. Then, membranes were incubated with HRP-conjugated goat anti-mouse IgG antibody (dilution with 1:4000 in PBS) at 37 °C for 1 h. At last, the substrate of 3,3*N*-diaminobenzidine tertrahydrochloride (DAB) was used to visualize the reaction result. 

Indirect immunofluorescence assay (IFA) was used to identify the reactivity of mAb 3D2 with DAstV-1 virus. Briefly, 70–90% confluent BHK-21 cells were transfected with the DNA-launched infectious clone of DAstV-1 or recombinant mutant plasmids. At 48 h post-transfection (hpt), the cells were fixed with an icy mixture of acetone-formaldehyde (1:1 *v/v*) for 20 min and followed by transparent with 0.2% Triton X-100 in PBS for 3 min. After cells being blocked with PBS buffer containing 1% bovine serum albumin for 1 h, mAb 3D2 (dilution with 1:1000), mouse polyclonal antibody (dilution with 1:500) or SP2/0 supernatant (dilution with 1:50) were added as the primary antibodies. After washing three times with PBST buffer, the secondary antibody, FITC-conjugated goat anti-mouse IgG (dilution with 1:50) was added and incubated at 37 °C for 1 h in the dark. Finally, the stained cells were observed using a fluorescence microscope (Leica AF6000).

### 2.3. Neutralization Assay for mAb 3D2 Against DAstV-1

Virus neutralization assay was used to detect the neutralizing activity of mAb 3D2 against DAstV-1 by IFA. Briefly, 100 μL of serial diluted mAb 3D2 samples (the initial dilution of 1:10; and 2-fold dilutions to 1:160) were incubated with at 1 MOI (multiplicity of infection) of DAstV-1 for 2 h at 37 °C, respectively. Then, 200 μL virus-mAb mixture was added into DEF monolayer cells. At 48 h post infection (hpi), the cells were observed and analyzed by IFA.

### 2.4. Identification of the B-Cell Linear Epitope to 3D2

To preliminarily identify the epitope that mAb 3D2 can recognize, three overlapping fragments of the DAstV-1 *ORF2* gene were amplified with specific primers (Table 2) and cloned into plasmid pGEX-6p-1 for construction of the recombinant plasmids ORF2-A, ORF2-B, and ORF2-C. All truncated GST-fusion proteins were expressed and purified, and subsequently were analyzed by dot blot analysis using mAb 3D2 or anti-GST mAb, respectively. 

Based on the dot blot detecting results, five fragments (ORF2-I, ORF2-II, ORF2-III, ORF2-IV, and ORF2-V) were designed to further determine the position of the epitope (Table 2). The recombinant proteins were detected by dot blot assay. Finally, six fragments (ORF2-1, ORF2-2, ORF2-3, ORF2-4, ORF2-5, and ORF2-6) were designed to determine the accurate position of the epitope by cutting down the amino acids from both sides one by one (Table 2). The recombinant proteins were also detected by dot blot analysis. 

### 2.5. Cross-Reactivity of the mAb 3D2

To assess the conservation of the epitope among DAstV-1 (C-NGB, WF1201, DA06, DA07, DA08, and DA93), DAstV-2 (SL1, SL2, SL4, and SL5), DAstV-3 (CPH), and DAstV-4 (YP2), we further performed sequence alignment of the corresponding locations of the epitope in the ORF2 protein using Lasergene software (DNAStar). 

**Figure 1:**
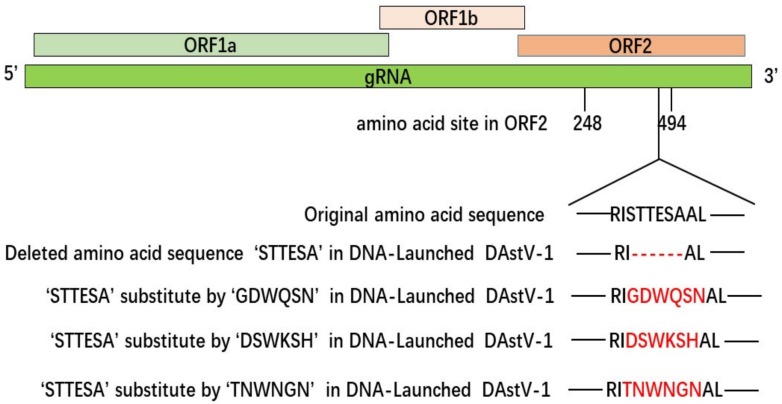
Schematic representation of the mutant DAstV-1 used in this study.

Subsequently, we constructed different mutations in DNA-Launched clone of DAstV-1, and they were the clone deleted epitope ‘STTESA’ followed by the substitution of amino acids in the epitope motif location with ‘GDWQSN’, ‘DSWKSH’, ‘TNWNGN’, ‘STAETG’, and ‘STTETG’, respectively (Figure 1, Table 3). In detail, we firstly amplified the upstream (primers of U-F and U-R) and downstream (primers of D-F and D-R) of the products, respectively. Then, based on the U-F primer, a series of reverse primers (UF-1R, UF-2R, UF-3R, and UF-4R) was used for PCR amplification. At the same time, based on the D-R primer, a series of forward primers (DR-1F, DR-2F, DR-3F, and DR-4F) were used for PCR amplification. These PCR products were finally fused to obtain the mutations mentioned above. When reaching 70–90% confluence, BHK-21 cells were respectively transfected with those mutant plasmids and analyzed by IFA at 48 hpt.

## 3. Results

### 3.1. Reactivity Analysis of mAb 3D2

**Figure 2:**
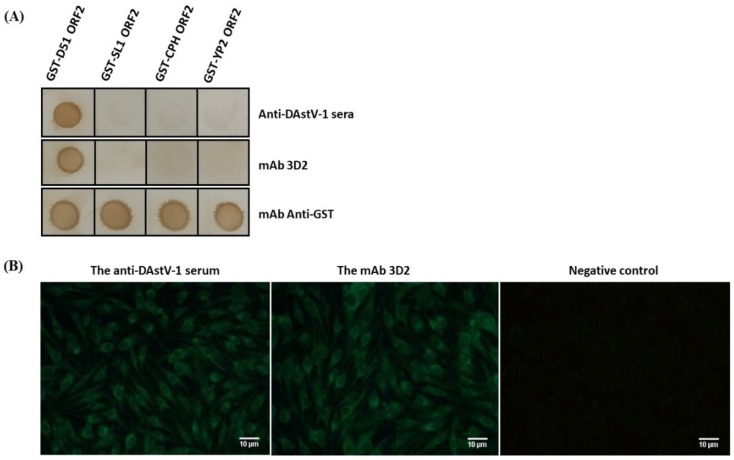
Reactivity analysis of monoclonal antibody (mAb) 3D2. (**A**) The specificity of the mAb 3D2 to the recombinant glutathione S-transferase tagged ORF2 (GST-ORF2) proteins of the four serotypes of DAstV was detected by dot blotting. The mAb 3D2 specifically reacted with GST-D51 ORF2, while not with GST-SL1 ORF2, GST-CPH ORF2 and GST-YP2 ORF2; (**B**) Reactivity of mAb 3D2 using indirect immunofluorescence assay (IFA).

Dot blotting results showed that mAb 3D2 specifically reacted with GST-D51 ORF2, while did not react with GST-SL1 ORF2, GST-CPH ORF2, and GST-YP2 ORF2 (Figure 2A). The IFA result revealed that mAb 3D2 could recognize the native-form of the ORF2 protein, whereas could not recognize GST-SL1 ORF2, GST-CPH ORF2, and GST-YP2 ORF2 (Figure 2B). Those results suggested that mAb 3D2 had good specificity with ORF2 protein of DAstV-1 and did not react with the ORF2 proteins of other serotypes of DAstV.

### 3.2. Neutralization Analysis 

**Figure 3:**
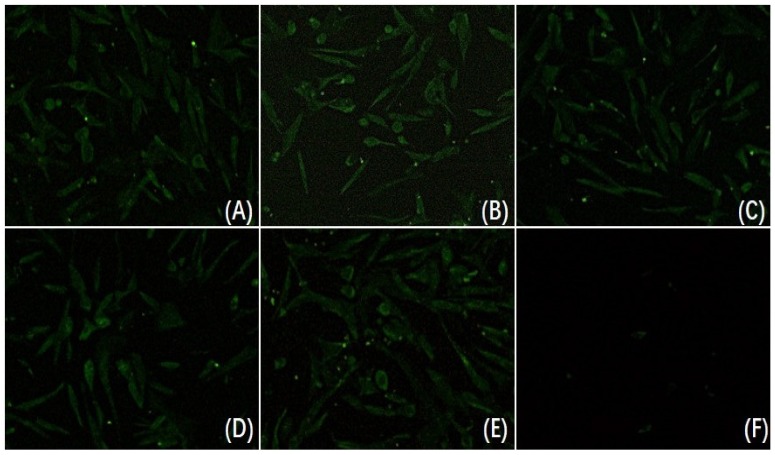
Neutralization assay of mAb 3D2. (**A**) The duck embryo fibroblasts (DEF) cells were infected with virus-mAb mixture (*v:v*, 1:1; dilution of mAb 3D2: 1:10); (**B**) the DEF cells were infected with virus-mAb mixture (*v:v*, 1:1; dilution of mAb 3D2: 1:20); (**C**) the DEF cells were infected with virus-mAb mixture (*v:v*, 1:1; dilution of mAb 3D2: 1:40); (**D**) the DEF cells were infected with virus-mAb mixture (*v:v*, 1:1; dilution of mAb 3D2: 1:80); (**E**) the DEF cells were infected with virus-mAb mixture (*v:v*, 1:1; dilution of mAb 3D2: 1:160); (**F**) the negative control.

The virus-mAb mixture, a series of diluted mAb 3D2 incubated with DAstV-1 for 1 h at 37 °C, was adsorbed in the DEF cell for 2 h and fresh DMEM with 2% FBS was added after washed with PBS. The IFA result showed that there was still strong green fluorescence whether in the maximum concentration (dilution with 1:10) or the minimum concentration (dilution with 1:160) of the mAb 3D2 treatment group (Figure 3). It indicated that the mAb 3D2 had no neutralizing activity to DAstV-1. 

### 3.3. The Accurate Position of the Linear Epitope in DAstV-1 ORF2 to mAb 3D2

**Figure 4:**
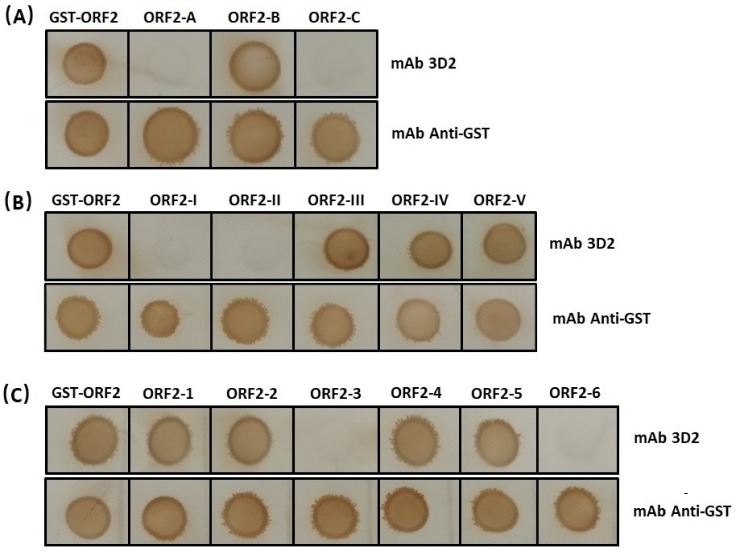
Screening of the B-cell linear epitope against mAb 3D2. (**A**,**B**) Preliminary screening the epitope using dot-blot with mAb 3D2 and anti-GST antibody being the primary antibody; (**C**) the accurate confirmation of the linear epitope by reducing the amino acids either from the N-terminal or the C-terminal.

A total of three overlapping GST-fused fragments (ORF2-A, ORF2-B, and ORF2-C) of DAstV-1 ORF2 protein were successfully expressed. Dot blotting results showed that mAb 3D2 reacted with ORF2-B, while did not react with ORF2-A and ORF2-C (Figure 4A). Subsequently, five overlapping GST-fused fragments (ORF2-I, ORF2-II, ORF2-III, ORF2-IV, and ORF2-V) were successfully expressed and dot blotting results showed that mAb 3D2 reacted with ORF2-III, ORF2-IV, and ORF2-V, while not with ORF2-I and ORF2-II (Figure 4B). Therefore, we initially inferred that the epitope against mAb 3D2 was within the amino acids of ^452^RISTTESAAL^461^ in DAstV-1 ORF2 protein. 

**Figure 5:**
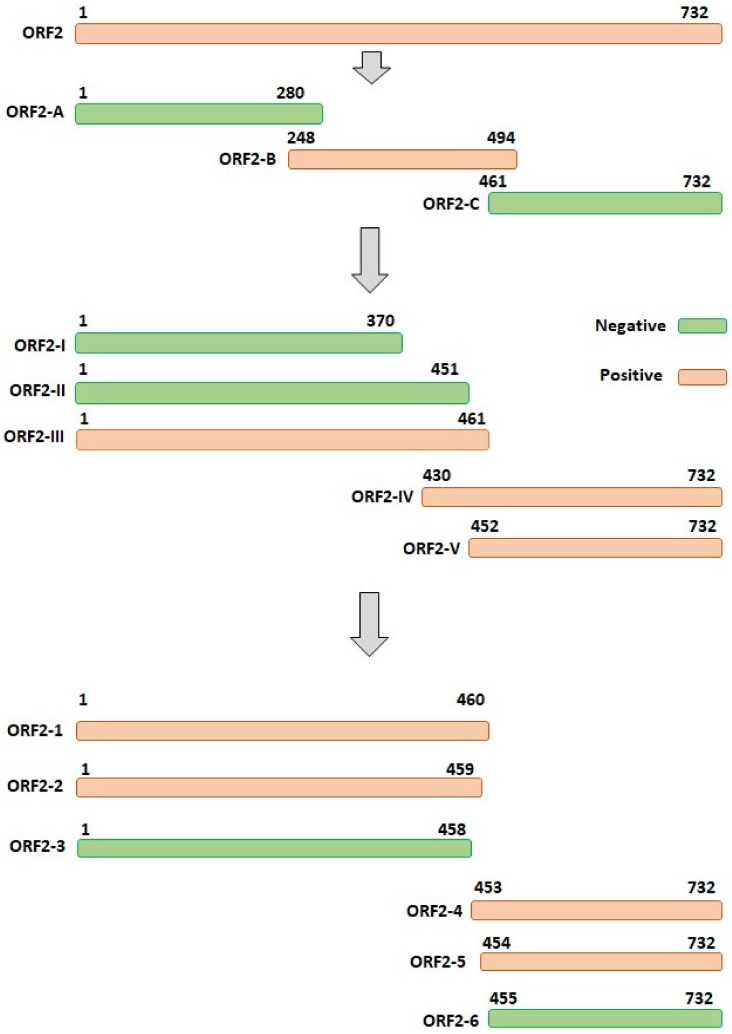
Schematic diagram of the dot blot test.

Based on the motif of ^452^RISTTESAAL^461^, we reduced the amino acids one by one from both the N-terminal and the C-terminal to determine the accurate epitope (Table 2). A total of six overlapping GST-fused fragments (ORF2-1, ORF2-2, ORF2-3, ORF2-4, ORF2-5, and ORF2-6) of DAstV-1 ORF2 protein were successfully expressed. Dot blot assay revealed that mAb 3D2 did react with ORF2-1, ORF2-2, ORF2-4, and ORF2-5, while did not react with ORF2-3 and ORF2-6 (Figure 4C). Based on the dot blot analysis results, the accurate position of the epitope recognized by mAb 3D2 was deduced at ^454^STTESA^459^ in the ORF2 protein of DAstV-1 (Figure 5).

### 3.4. Epitope Mapping of the mAb 3D2 

**Figure 6:**
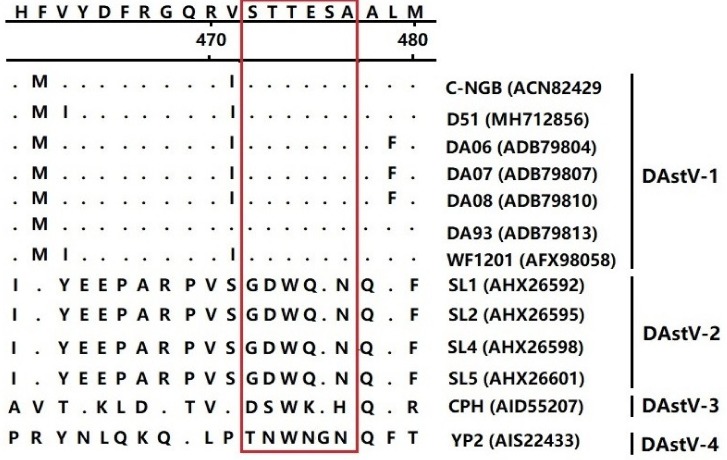
Sequencing alignment of DAstV strains around the epitope-coding region of the ORF2 protein. DAstV-1 C-NGB strain sequences are shown at the top; the dot indicates identical amino acids of the epitope. The amino acid in the rectangle indicates the epitope.

To identify whether the ^454^STTESA^459^ epitope was conserved in the ORF2 protein among different serotypes of DAstV, we aligned all the ORF2 sequences of DAstV-1, DAstV-2, DAstV-3, and DAstV-4 in GenBank. The result showed that the amino acid sequences in the epitope motif were conserved among all the DAstV-1 strains, but were quite different from the amino acids in the corresponding regions of other DAstV serotypes (Figure 6), indicating that the motif represented a conserved epitope on the ORF2 protein of DAstV-1.

**Figure 7:**
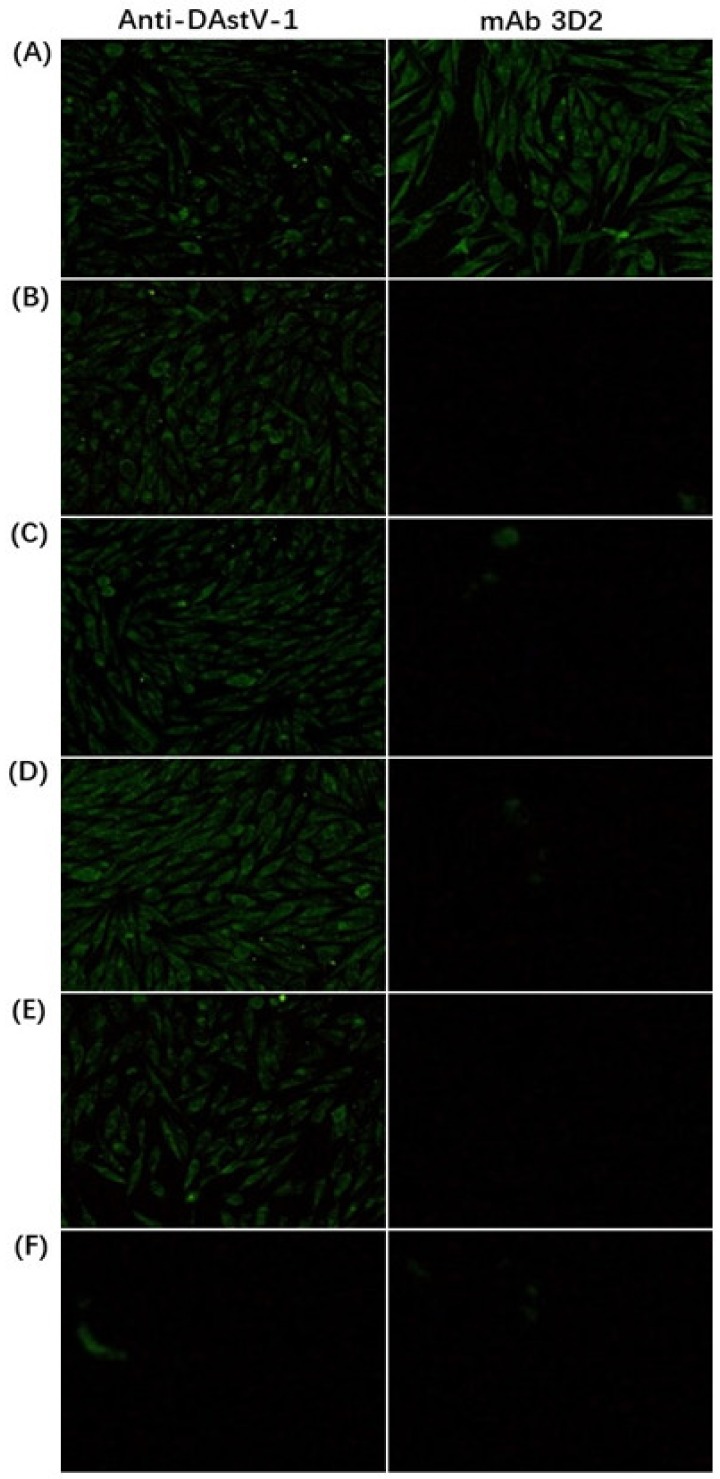
Cross-reactivity analysis in baby hamster kidney (BHK -21) cells by IFA with the DAstV-1 positive sera and the mAb 3D2. (**A**) BHK-21 cells infected by DNA-launch infection clone of DAstV-1; (**B**) BHK-21 cells infected by DAstV-1 with the deleted epitope; (**C**) BHK-21 cells infected by the epitope substituted with ‘GDWQSN’; (**D**) BHK-21 cells infected by the epitope substituted with ‘DSWKSH’; (**E**) BHK-21 cells infected by the epitope substituted with ‘TNWNGN’; (**F**) the normal BHK-21 cells.

To further verify the epitope of mAb 3D2, using the DNA-launched infectious clone of DAstV-1, epitope ^454^STTESA^459^ was firstly deleted by primer mutation, and then respectively substituted by ^454^GDWQSN^459^ of DAstV-2 ORF2, ^454^DSWKSH^459^ of DAstV-3 ORF2 and ^454^TNWNGN^459^ of DAstV-4 ORF2. IFA results showed that mAb 3D2 reacted with DAstV-1 molecular strains, while not with the mutant ones (Figure 7).

## 4. Discussion

DAstV-1 infection causes fatal hepatitis to ducklings [13,18] and is ubiquitous in China [30,31]. As the most prevalent serotype, the overall mortality in 1- to 2-week-old ducklings infected by DAstV-1 was 50%, and livers in the affected ducklings displayed typical multiple and widespread hemorrhagic lesions [13,32]. As an effective antiviral agent and differential diagnostic agent, mAbs of HAstV and CAstV had been used to diagnosis [33,34]. However, there is still a lack of new classes of antibodies for diagnosis of DAstV-1 infection. From a genetic point of view, *ORF1b* of astrovirus was the least divergent whereas *ORF2* appeared to be the most divergent among the different ORFs [35]. Moreover, as viral capsid protein, ORF2 protein serves as the major candidate antigen for vaccine preparation or virus serological diagnosis [36]. In this study, mAb 3D2 against DAstV-1 ORF2 protein was firstly prepared, and IFA showed that mAb 3D2 specifically recognized DAstV-1-transfected BHK-21 cells (Figure 2). 

In terms of diagnostic and disease monitoring, epitopes can be used to monitor the response to the particular virus strain during infection. B-cell epitopes could bind to corresponding receptors in humoral and cellular immune responses and contribute to the specific antibody production [37]. Well-defined epitopes against mAbs will contribute to benefit from the antigen structure study and vaccine preparation [38,39,40,41]. Therefore, it is imperative to screen B-cell epitopes in epitope-based diagnosis kits. Among the common antigen-antibody specificity methods such as enzyme-linked immunosorbent assay (ELISA) and Western blot, dot blot is a low-complexity and time-saving technique that is widely used for peptide screening [29,42]. Using dot blotting, the epitope ^454^STTESA^459^ against the mAb 3D2 was identified in the present study. To the best of our knowledge, this was the first accurate epitope identified in DAstV-1.

As the major protective antigenic protein, the ORF2 protein is hypervariable in AstVs [36,43]. The epitope motif identified in this study was conserved among all the DAstV-1 strains, but was obviously different from the strains of DAstV-2, DAstV-3, and DAstV-4 (Figure 6). To date, only DAstV-1 can be cultured and passaged in cells [28], while other serotypes of DAstV can only be passaged by ducklings [15,16,17]. Reactivity analysis showed that the mAb 3D2 could only react with the epitope ^454^STTESA^459^ in DAstV-1 ORF2, while not with ^454^GDWQSN^459^ in DAstV-2 ORF2, ^454^DSWKSH^459^ in DAstV-3 ORF2, and ^454^TNWNGN^459^ in DAstV-4 ORF2 (Figure 7). The mAb 3D2 had no cross-reactivity with the other serotypes of DAstV, indicating that it could be used in the DAstV-1 infection diagnosis.

## 5. Conclusions

In conclusion, a conserved linear B-cell epitope in DAstV-1 ORF2 protein was identified for the first time. The 3D2 was a highly specific mAb without neutralizing activity to DAstV-1, which might play a significant role in the development of a discriminating diagnostic kit for DAstV-1 infection.

## Figures and Tables

**Table 1:** Primers for expression the complete ORF2 protein of the four serotypes of duck astrovirus (DAstV).

Primers	Sequence (5′→3′)	AA Position in ORF2 Protein
D51-ORF2-F	CCGGAATTCATGGCTGGTGAGGCCCTTG	1–732
D51-ORF2-R	CCGCTCGAGCTACTCGGCGTGGCCGCGGCT	
SL1-ORF2-F	CCGGAATTCATGGTGGCGGCGATGGCCGA	1–727
SL1-ORF2-R	CCGCTCGAGCTACTCGGCGTGGCCTCCAC	
CPH-ORF2-F	CCGGAATTCATGGCCGATAAGGCTGTTGT	1–746
CPH-ORF2-R	CCGCTCGAGCTACTCGGCGTGGCCACGGC	
YP2-ORF2-F	CCGGAATTCATGACTGGGGCCACCCCAA	1–728
YP2-ORF2-R	CCGCTCGAGCTACTCGGCGTGGCCGCGGC	

Note: EcoR I and Xho I restriction enzyme sites were introduced at the 5′ end of the primers with underlined letters.

**Table 2:** Primers for identification of the linear epitope in DAstV-1 D51 strain.

**Primers**	**Sequence (5′** **→3′)**	**AA Position in ORF2 Protein**
ORF2-AF	CCGGAATTCATGGCTGGTGAGGCCCTTG	1–280
ORF2-AR	CCGCTCGAGAGTGTAGTTATTTGAAATC	
ORF2-BF	CCGGAATTCACCAGTGTTCTCTTTTTGGT	248–494
ORF2-BR	CCGCTCGAGAGACTTCTGTCTGCCATTGT	
ORF2-CF	CCGGAATTCCTAATGATTGCAGCTATACC	461–732
ORF2-CR	CCGCTCGAGCTACTCGGCGTGGCCGCGGCT	
ORF2-AF	CCGGAATTCATGGCTGGTGAGGCCCTTG	1–370
ORF2-IR	CCGCTCGAGGGCATCATTGGCAGCACCA	
ORF2-AF	CCGGAATTCATGGCTGGTGAGGCCCTTG	1–451
ORF2-IIR	CCGCTCGAGTTGACCACGAAAGTCATAAAT	
ORF2-AF	CCGGAATTCATGGCTGGTGAGGCCCTTG	1–461
ORF2-IIIR	CCGCTCGAGTAGTGCTGCTGATTCTGTG	
ORF2-IVF	CCGGAATTCTACTTGCCCTTGCCTCTCGC	430–732
ORF2-CR	CCGCTCGAGCTACTCGGCGTGGCCGCGGCT	
ORF2-VF	CCGGAATTCAGAATCAGCACCACAGAATC	452–732
ORF2-CR	CCGCTCGAGCTACTCGGCGTGGCCGCGGCT	
ORF2-AF	CCGGAATTCATGGCTGGTGAGGCCCTTG	1–460
ORF2-1R	CCGCTCGAGTGCTGCTGATTCTGTGGTGC	
ORF2-AF	CCGGAATTCATGGCTGGTGAGGCCCTTG	1–459
ORF2-2R	CCGCTCGAGTGCTGATTCTGTGGTGCTGA	
ORF2-AF	CCGGAATTCATGGCTGGTGAGGCCCTTG	1–458
ORF2-3R	CCGCTCGAGTGATTCTGTGGTGCTGATTC	
ORF2-4F	CCGGAATTCATCAGCACCACAGAATCAG	453–732
ORF2-CR	CCGCTCGAGCTACTCGGCGTGGCCGCGGCT	
ORF2-5F	CCGGAATTCAGCACCACAGAATCAGCAG	454–732
ORF2-CR	CCGCTCGAGCTACTCGGCGTGGCCGCGGCT	
ORF2-6F	CCGGAATTCACCACAGAATCAGCAGCACT	455–732
ORF2-CR	CCGCTCGAGCTACTCGGCGTGGCCGCGGCT	

Note: EcoR I and Xho I restriction enzyme sites were introduced at the 5′ end of the primers with underlined letters.

**Table 3:** Primers for mutation in the DNA-Launched infectious clone of DAstV-1.

Product Name	Primers	Sequence (5′→3′)
Upstream	U-F	GGAAGATCTGGAGGCTGTTGAACCG
	U-R	GATTCTTTGACCACGAAAGTCATAA
Downstream	D-F	GCACTAATGATTGCAGCTATACCAC
	D-R	TCCCCGCGGCTGCTGGCAGCTTGTTGT
Delete epitope	UF-1R	ATTTGCTTGTGGTATAGCTGCAATCATTAGTGCTGCACCACGAAAGTCATAAATCA
	DR-1F	CAGTATACACCACACATGATTTATGACTTTCGTGGTGCAGCACTAATGATTGCAGC
STTESA-GDWQSN	UF-2R	GTGCTGCTGATTCTGTGGTGCTATTACTCTGCCAATCACCGATTCTTTGACCACGAAAG
	DR-2F	TGACTTTCGTGGTCAAAGAATCGGTGATTGGCAGAGTAATGCACTAATGATTGCAGCTA
STTESA-DSWKSH	UF-3R	GGTATAGCTGCAATCATTAGTGCGTGGCTCTTCCATGAATCGATTCTTTGACCACGAAAG
	DR-3F	ATGACTTTCGTGGTCAAAGAATCGATTCATGGAAGAGCCACGCACTAATGATTGCAGCT
STTESA-TNWNGN	UF-4R	TGGTATAGCTGCAATCATTAGTGCATTGCCATTCCAGTTGGTGATTCTTTGACCACGAA
	DR-4F	ATGACTTTCGTGGTCAAAGAATCACCAACTGGAATGGCAATGCACTAATGATTGCAGCT

Note: Bgl II and Sac II restriction enzyme sites were introduced with the underlined letters.
